# Fast, easy and early (larval) identification of transparent mutant zebrafish using standard fluorescence microscopy

**DOI:** 10.12688/f1000research.22464.1

**Published:** 2020-08-10

**Authors:** Ralf Wenz, Emily Conibear, Laurence Bugeon, Maggie Dallman

**Affiliations:** 1 Life Sciences, Imperial College London, London, Greater London, SW7 2AZ, UK

**Keywords:** tra, nac, trab6/b6nacw2/w2, casper, Zebrafish, transparent, translucent, screening, iridophore

## Abstract

The availability of transparent zebrafish mutants (either *TraNac*: *tra ^b6/b6^; nac ^w2/w2 ^*or *casper: roy ^a9/a9^; nac ^w2/w2^*) for live imaging studies together with the ease of generating transgenic lines are two of the strengths of the zebrafish model organism. The fact that transparent *casper* ( *roy ^a9/a9^;nac ^w2/w2^)* and silver *nacre* ( *nac ^w2/w2^)* mutants are indistinguishable by eye at early stages (1-5 days post-fertilization; dpf) means many fish must be raised and later culled if they are not transparent. To identify translucent mutants early and easily at the early larval stage (≤5 dpf) before they are classified as protected animals, we developed a simple screening method using standard fluorescence microscopy. We estimate that this procedure could annually save 60,000 animals worldwide.

**Table:** 

Research highlights	
Scientific benefit	Early identification of *TraNac* I *casper* mutations in zebrafish larvae (5 dpf)
3Rs benefit	Early screening of zebrafish larvae could result in 60,000 fewer adult fish being raised and culled, annually worldwide. For each zebrafish mutant line, an approximate 75% reduction in animal use could be achieved.
Practical benefits	Fast, early and easy identification of transparent zebrafish larvae at 5 dpf.
	Reducing the number of animals raised by 75% concomitantly decreases the costs associated with animal husbandry
Current application	Screening *TraNac* / *casper* mutants in zebrafish larvae
Potential application	Automated screening of *TraNac* / *casper* mutants

## Introduction

**Figure 1.**
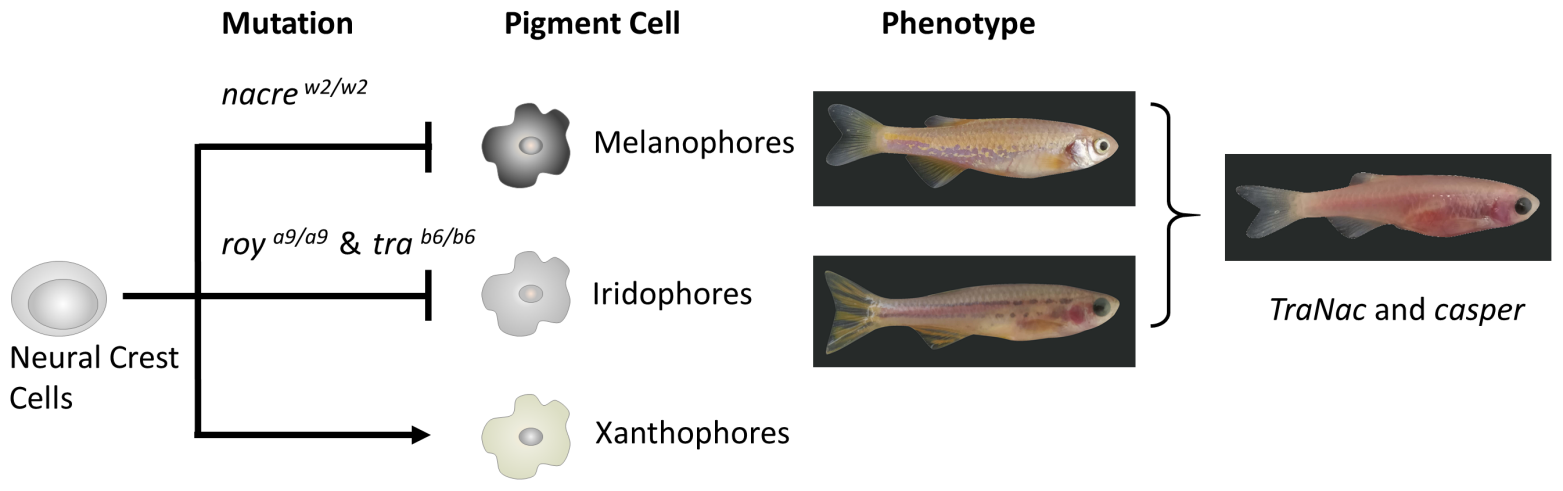
Zebrafish mutants, the affected pigment cells and corresponding phenotypes. The pattern characteristic of Zebrafish colouration depends on three pigment cell types: melanophores, xanthophores and iridophores ( Singh & Nüsslein-Volhard, 2015). Mutational inactivation of two of those chromatophores gives rise to transparent zebrafish ( *TraNac* and *casper)*. *TraNac* = *tra ^b6/b6^; nac ^w2/w2^* compound zebrafish mutants, *nac = nacre, roy = roy orbison, tra = transparent,* WT = wild-type.

The zebrafish is a very popular vertebrate model organism, being the second most commonly used animal species in Great Britain. Of the 1.72 million procedures in 2018 purely relating to the creation and breeding of genetically altered animals, 223,600 (13%) were zebrafish ( Home Office, 2019). This is because, amongst other beneficial features, one zebrafish female can produce several hundred eggs in a single clutch ( Lawrence, 2011). Moreover, zebrafish lend themsleves to live imaging even at later stages of development due to the availability of transparent mutants. These transparent mutants are homozygous compound mutants known as *TraNac* ( *tra ^b6/b6^;nac ^w2/w2^*), and *casper* ( *roy ^a9/a9^;nac ^w2/w2^*) ( Figure 1).

There are two mutations involved in changing the pigmentation of zebrafish. The first is *nacre* ( *nac ^w2/w2^*). *Nacre* mutants do not have a functional transcription factor encoded by *mitfa* and therefore lack melanophores ( Lister *et al.*, 1999). This results in a uniformly silvery coloured ‘ *nacre*’ zebrafish. The second mutation involved in pigmentation is *roy orbison* ( *roy ^a9/a9^*), or *roy* hereafter. *Roy* has the identical frameshift and premature stop codon as the mutation *transparent* ( *tra ^b6/b6^*), which will be referred to as *tra* ( D’Agati *et al.*, 2017). Both *roy* mutants ( Ren *et al.*, 2002) and *tra* mutants ( Krauss *et al.*, 2013) have an aberrant mitochondrial inner membrane protein 17 (Mpv17 protein) and therefore lack iridophores ( D’Agati *et al.*, 2017). This results in zebrafish that have no silver pigment but instead black spotted melanocytes. If both mutations, *nac* and *roy / tra*, are present and homozygous, the fish will lack melanophores and iridophores and are thus transparent ( Figure 1).

**Figure 2.**
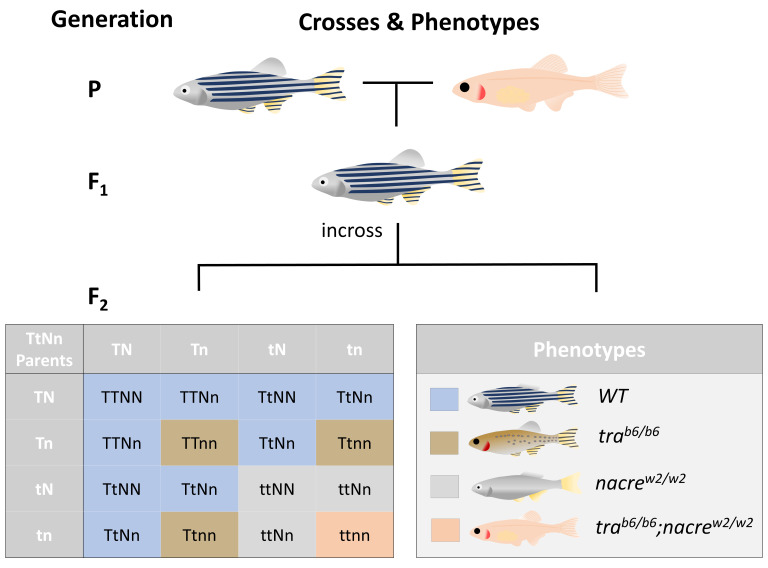
Theoretical ratios of crosses between wild-type and transparent zebrafish. The earliest possible transparent phenotype after a wild-type and *TraNac* fish have been bred (P generation) is the second generation (F2). However, at that point, only 6.25% of all fish will theoretically be transparent, due to the genetic inheritance pattern of both tra and nacre alleles being passed on in their mutated form ( *tra ^b6^* & *nacre ^w2^*). As indicated in the Punnet square, there are 16 possible combinations of genes (T indicating functional tra allele and t represents mutated *tra ^b6^*, while N indicates functional nacre and n mutated *nacre ^w2^*). The 16 possible combinations can result in 4 different phenotypes WT pattern, *tra* pigmented pattern, silvery *nacre*, transparent *TraNac* with the associated ratio of 9:3:3:1. *nac* = *nacre*, *tra* = *transparent*, WT = wild-type.

When one requires transparent *TraNac* or *casper* zebrafish to also express a specific transgene, the transgenic line of interest - commonly created on a wild-type (WT) background – is crossed with the transparent mutant line. The first generation will have a WT pigmentation phenotype. The incrossed second generation will be a mix of WT, silver *nac ^w2/w2^*, spotted *tra ^b6/b6^*, and transparent mutants ( *TraNac* or *casper*) in a ratio of 9:3:3:1 ( Figure 2). While it is possible to identify WT and *tra* zebrafish before six days post fertilization (dpf) by simply screening for melanocyte pigmentation, it is currently not possible to distinguish between *nacre* and transparent *TraNac* / *casper* zebrafish before 6 dpf. Therefore all transparent looking 5 dpf fish ( *nacre* and *TraNac* / *casper*) are currently raised to an age at which they can be distinguised, which is about 2 months post-fertilisation. At this point, not needed *nacre* fish can be culled. This means that even after removing all pigmented embryos before 5 dpf, ~75 % of the remaining second generation still must be culled at a later date. Therefore, a method that could identify transparency before 6 dpf; the stage at which they become protected animals under the Animals (Scientific Procedures) Act, 1986 would potentially reduce the number of protected animals culled every year by thousands.

We have identified a way to screen for *TraNac* and *casper* mutants at early stages using conventional stereo-microscopy. This new approach has two major advantages: firstly, this approach allows the early and easy identification of transparent zebrafish for experiments; and secondly, crossing WT zebrafish onto transparent backgrounds will not require any culling of unwanted intermediate *nacre* fish of the second generation at a legally protected age. Therefore, this approach could save 60,000 adult fish worldwide every year (the detailed analysis of this metric follows in the dicussion).

## Methods

### Ethical statement

Zebrafish were maintained using standard practices and all procedures conformed to the Animals (Scientific Procedures) Act, 1986 of Government of the United Kingdom as well as the Directive 2010/63/EU of the European Parliament. Animals were maintained under UK Home Office project licence number P5D71E9B0. All efforts were made to minimize animal suffering by daily surveillance of animal health and water conditions, enriching the environment using live feed, by not performing invasive procedures that may in any way harm the animal and by reducing the number of animals necessary.

### Animal husbandry

Rearing and maintenance of the WT, *TraNac, nacre* and *casper* fish was carried out at 28.5°C on a 14 h light/10 h dark cycle. AB fish strain of both sexes were used and were sourced locally from Imperial College Central Biological Services. The system water was derived from deinoised water reconstituted with sodium chloride salt to a final conductivity of 750 μS ± 50, while pH levels were kept within boundaries of 7.0 ± 0.2. Fish were housed in 3-litre see-through polycarbonate tanks of the Aquatic Habitats Z-Hab System (MBKI, Nottingham, UK) with a density of around 5–7 fish per litre. Feeding of fish was done according to stages twice a day, once in the morning and once in the evening: 6 dpf – 8 dpf fish were fed with ZM000 by ZM systems, 9 dpf – 14 dpf fish were fed with ZM100 (ZM systems), 15 dpf – 2 months post-fertilization old fish were fed with ZM200 (ZM systems), while any older adults were fed with pellet food by Hikari Tropical. As part of the environmental enrichment, adult fish were fed live *Artemia salina* once a day in the morning.

### Screening procedure

Three experiments with two experimental groups each were done. We compared the correct identification of *TraNac* vs *nacre* fish, as they are indistinguishable by eye at 5 dpf. In the *TraNac* groups of the three separate experiments were 16, 9, and 12 fish, respectively; while in the *nacre* groups of the three separate experiments were 19, 13 and 15 fish, respectively. We had, using power calculations, determined that 15 adult fish per group would render 90% power at a 0.05 significance level, a standard deviation of 2, and a difference in mean of 2.5. In this study, we obtained on average 14 fish per group. This, however, still rendered a 88% power and which still is accepted as scientifically valid according to the documentation of the NC3Rs’ Experimental Design Assistant ( NC3Rs EDA, 2020).

**Figure 3.**
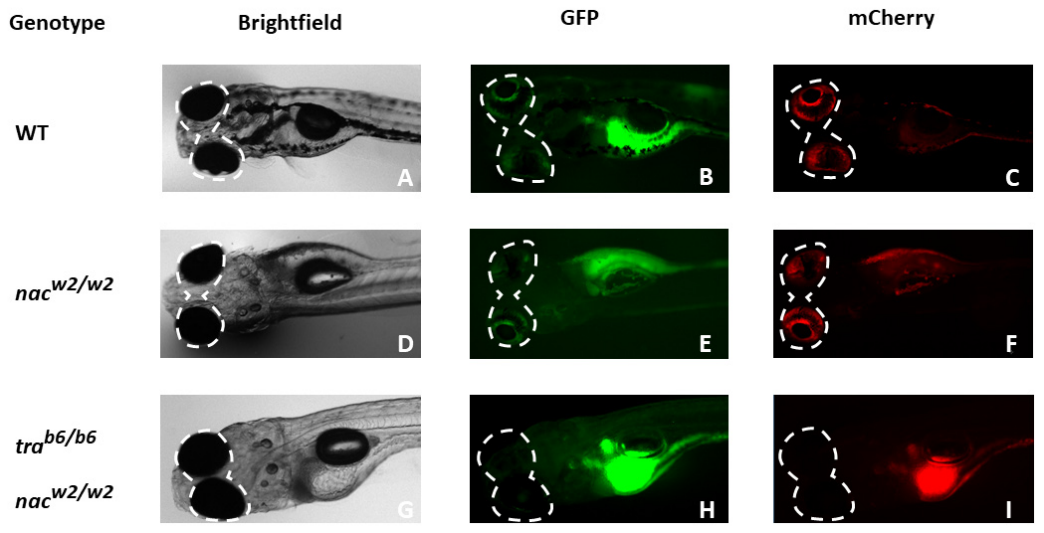
Larval zebrafish screening at 5 dpf using fluorescent microscopy and investigation of eye autofluorescence caused by iridophores. Using different channels (brightfield, GFP and mCherry), different patterns of colouration in the zebrafish eye become apparent between *TraNac* zebrafish larvae ( **I**), in contrast to both *nac* ( **E**), and WT ( **C**) larvae. Although both fluorescent channels, GFP and mCherry appear to be equally useful for screening for eye pigmentation, by experience, the red fluorescent mCherry channel is easier for distinguishing in practice. *nac = nacre, roy = roy orbison, tra = transparent,* WT = wild-type.

**Figure 4.**
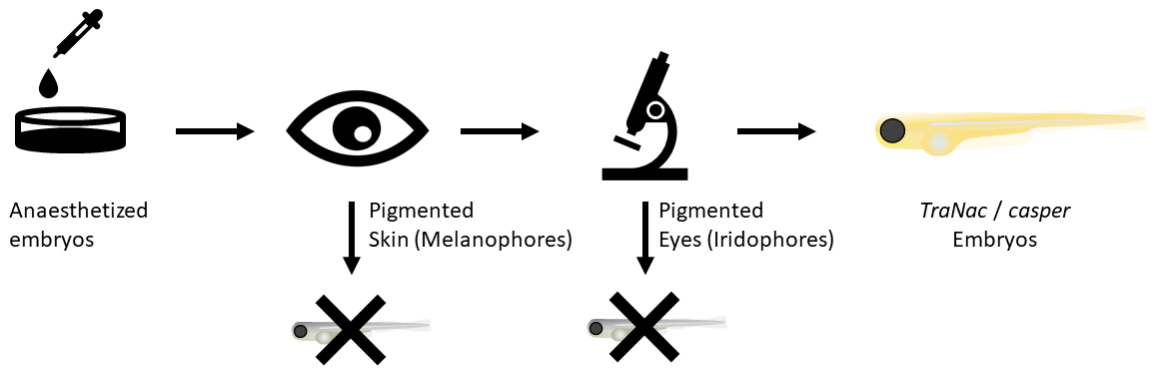
Screening procedure. Fish are anaesthetized in 4.2% MS-222 (168 µg/mL). Thereafter simple visual screening of larvae allows WT and *tra* fish to be discarded. The next step is fluorescent microscopy screening using the mCherry filter for different colour patterns in the eyes of the fish (see Figure 3). If *TraNac* or *casper* fish are desired, one screens for fish without any eye pigmentation. *TraNac* = *tra ^b6/b6^; nac ^w2/w2^* zebrafish mutants.

Zebrafish from 0 days post-fertilization (dpf) to 5 dpf were reared in Petri dishes in system water with with 3x10 ^-5^% methylene blue. For anesthesia, fish were transferred into a new Petri dish containing 4.2% (168 µg/mL) MS-222 (Sigma, E10521-50G) in system water with 3x10 ^-5^% methylene blue. Fish were screened by observing different fluorescent patterns of the eyes as illustrated in Figure 3 and Figure 4. A Leica M205 FCA stereomicroscope using a Leica DFC7000 T camera, the Leica LAS X software, and the Leica EL6000 external light source for fluorescence excitation was used for all experiments. The filters used were the Leica ET mCherry (Article Number: 10450195; Excitation nm: ET560/40x; Emission nm: ET630/75m) as well as the ET GFP (Article Number: 10447408; Excitation nm: ET470/40x; Emission nm: ET525/50m). Once screened according to phenotype, fish were transferred to a new Petri dish containing only system water and methylene blue. The screening procedure takes, depending on practice, approximately 5-10 minutes, per dish of 100 fish.

To determine the screening efficiency of the above procedure (see also Figure 3 and Figure 4), the screened 5 dpf fish were allowed to develop to the adult stage, the stage at which skin pigmentation can be clearly seen ( Figure 1). If adult fish had silver pigments in their skin they were identified as *nacre* fish, and if they had neither silver pigments nor melanocytes in their skin they were identified as *TraNac* fish. Fish were recorded as correctly screened at 5 dpf if the identified 5 dpf phenotype matched the phenotype at the adult stage.

## Results

We showed that *TraNac* and *casper* fish do not have autofluorescence in their eyes, when subject to fluorescence microscopy in the mCherry channel, in contrast to WT fish. Through this finding we were able to develop a simple two-step process to identify transparent *TraNac* or *casper* zebrafish as outlined in Figure 4. First, after anaesthetising the fish, embryos that were observed by eye to have black pigments were discarded. These were either WT or *tra* mutants that still produce melanophores. Subsequently, using a fluorescent stereo-microscope with an mCherry filter, fish that did not have visible red eyes (see Figure 3, I) were identified. Those fish with visible red eyes using the mCherry filter were *nacre* mutants and would develop iridophores in the future ( Figure 3, C & F). Of note, while iridophores are already present at 3 dpf in the eyes of zebrafish ( Gur *et al.*, 2018), screening at 5 dpf was found to be easier.

Using this screening procedure, we were able to correctly identify ~99% of zebrafish embryos at 5 dpf, either *nacre ^w2/w2^* or *TraNac* ( Table 1). In three separate screening experiments (n = 84 fish) only one fish was wrongly identified at 5 dpf. This was confirmed by observing their pigmentation pattern at the adult stage. Similarly, *casper* zebrafish, which carry the same mutations as *TraNac* fish ( D’Agati *et al.*, 2017) can be screened with the same methodology. This screening method allows the identification of fully transparent zebrafish mutants before 6 dpf, the age at which they become protected animals under the Animals (Scientific Procedures) Act, 1986.

**Table 1.** Success rate of different screens for either *nacre* or *TraNac* zebrafish.

Experiment number	Phenotype screened for	Total (n)	Correct identification (n)	Incorrect identification (n)	Success ratio (%)
*Experiment 1*	*nacre*	19	19	0	100%
	*TraNac*	16	16	0	100%
*Experiment 2*	*nacre*	13	13	0	100%
	*TraNac*	9	9	0	100%
*Experiment 3*	*nacre*	15	14	1	93%
	*TraNac*	12	12	0	100%
Total		84	83	1	**99%**

In three separate experiments, fish from three separate clutches were screened for either *TraNac* or *nacre* phenotype. As a result, six screens for either *TraNac* or *nacre* zebrafish were performed. Screening for the desired zebrafish phenotype was done at 5 days post-fertilization and successful identification was assessed ≥ 2 months post fertilization. *TraNac* = *tra ^b6/b6^; nac ^w2/w2^* zebrafish mutants.

## Discussion

The method presented herein could lead to many thousands of animals not being culled after the age at which they become legally protected animals under the Animals (Scientific Procedures) Act, 1986. We estimate that every two years around 120,000 fish worldwide could be saved. This is based on two approximations: (1) We carried out a literature review which identified about 3% of labs using a mutation that is involved in making *TraNac* / *casper* zebrafish. We searched the online database Scopus, for articles published in the year 2018 with the following keywords and Boolean operators: zebrafish OR danio rerio AND adult. To obtain a managable number of papers we further narrowed down the search to only return papers in the subject area of ‘Immunology and Microbiology’. Of the 527 papers we could access 509. We found that about 2.9% (15 papers) used zebrafish with a *roy, tra* or *nacre* mutation. (2) A recent estimate by the NC3Rs states that there are about 3250 institutions in the world that use zebrafish ( Lidster *et al.*, 2017).

Taking the two approximations together with common husbandry practices, we can therefore make a reasonable estimate about the number of fish that are culled unneccessarily every year. If about 3% of all 3250 institutions use *TraNac* / *casper* zebrafish, that means that there are ~100 institutions that keep these fish. In our lab we keep 15 transgenic lines on a transparent background, but in the following we will assume most labs only keep 10. On average per transgenic line we keep 40 fish. To establish one tank with 40 *TraNac* or *casper* zebrafish about 120 non-transparent *nacre* fish would be culled (see Figure 2 for Punnet square and resultant ratio of 9:3:3:1). This means that in one lab alone, to establish 10 transgenic lines of transparent fish, 1,200 fish would be culled. Since there are roughly 100 institutions that keep transparent zebrafish, the total number of fish that would be culled is about 120,000. Further, since it is common practice to outcross the lines every 2 years onto a WT background to enrich genetic diversity, 120,000 fish that would need to be culled are generated every two years for breeding purposes alone.

It is likely that a large fraction of these 120,000 fish could be saved in the future, because the uptake of this method is simple and the barriers are so low. The microsopy is easy, fast, and inexpensive. In fact, by implementing this method significant long term cost savings are likely, as 75% less fish need to be raised to adulthood. Besides these practical benefits, this approach also has several scientific benefits. It is now possible to identify *TraNac* / *casper* mutants early in development, allowing one to study the downstream impact of these mutations while having siblings from the same parental clutch, which would have previously been impossible.

In conclusion, the method presented allows for fast, early and easy identification of transparent ( *TraNac* and *casper*) zebrafish and could lead to 60,000 adult fish being saved every year worldwide.

## Data availability

### Underlying data

Original microscopy image files from Figure 3 are provided in a TIF format. To view these files, they should be imported into an appropriate image processing program such as FIJI ( Schindelin *et al.*, 2012).

Zenodo: Fluorescent microscopy images of larval zebrafish of either TraNac, Nacre or WT background. http://www.doi.org/10.5281/zenodo.3813755 ( Wenz, 2020)

This project contains the following underlying data:

- Nacre_5dpf.tif
- TraNac_5dpf.tif
- WT_5dpf.tif

Data are available under the terms of the Creative Commons Attribution 4.0 International license (CC-BY 4.0).
