# Supplementary material for: Fast, easy and early (larval) identification of transparent mutant zebrafish using standard fluorescence microscopy
Source: F1000Res. 2020 Aug 10;9:963. [Version 1] doi: 10.12688/f1000research.22464.1 (PMC7475958; doi:10.12688/f1000research.22464.1)
Supplement: Peer review history

# Peer review: Reviewer response for version 1

**DOI:** 10.5256/f1000research.24792.r69153

**Published:** 2020-09-04

**Authors:** Robert Hindges

**Affiliations:** 1 Centre for Developmental Neurobiology, King's College London, London, UK

In this article, Wenz and colleagues present a simple and straight-forward method to screen zebrafish larvae at early stages, in order to identify different pigmentation mutant genotypes. The screening method is based on autofluorescence signal in the eyes of the larvae, visible in the red channel. This therefore abolishes the need to keep larvae longer than 5 days post fertilisation and raise them until adult stages in order to sort out the required genotype.

 Overall, I think this is an excellent approach, which will be helpful for the scientific community using these pigmentation mutants for their imaging experiments.

 I have some minor comments: 

 Legend to Figure 1: as the genotypes are explained for TraNac I would suggest to also include the genotype description for casper.

 Figure 3 has no scale bars

 Page 4, last paragraph before Methods: Typo “discussion”

 Page 5, last line of first paragraph: missing space between “animaland”

 Legend to Figure 4: the statement to “screen for fish without any eye pigmentation” is misleading, as these larvae have still pigmented eyes (in contrast for example to the Crystal mutant (roy, nacre, alb mutant), not mentioned here). It should rather say: “screen for fish without autofluorescence signal in the eyes.

 Possible limitation of this screening method for transgenic lines having red-fluorescent reporters expressed in the eye could be discussed.

 Although I appreciate the motivation of the authors to give an estimated number of saved adult fish per year, there are a lot of assumptions made. In addition, only a particular subject (Immunology & Microbiology) is used. I think it would be helpful to know the concrete number of adult fish saved in the authors’ laboratory over the last year in addition to the more speculative numbers based on Scopus searches.

Are the 3Rs implications of the work described accurately?

Yes

Is the work clearly and accurately presented and does it cite the current literature?

Yes

If applicable, is the statistical analysis and its interpretation appropriate?

Not applicable

Are all the source data underlying the results available to ensure full reproducibility?

Yes

Is the study design appropriate and is the work technically sound?

Yes

Are the conclusions drawn adequately supported by the results?

Yes

Are sufficient details of methods and analysis provided to allow replication by others?

Yes

Reviewer Expertise:

Zebrafish models, imaging, neuroscience, development, axon guidance, visual system, synapse, cell adhesion molecules

I confirm that I have read this submission and believe that I have an appropriate level of expertise to confirm that it is of an acceptable scientific standard.

# Peer review: Reviewer response for version 1

**DOI:** 10.5256/f1000research.24792.r70416

**Published:** 2020-09-02

**Authors:** Paul c. Evans

**Affiliations:** 1 Department of Infection, Immunity and Cardiovascular Disease, Medical School, University of Sheffield, Sheffield, UK

1. Figure 3. There is autofluorescence from the abdomen. Is this the yolk sac? Please label this.
2. Is there a complication with fluorescent transgenic embryos? I imagine that most lines will not have altered fluorescent eyes but perhaps this can be commented on.

Are the 3Rs implications of the work described accurately?

Yes

Is the work clearly and accurately presented and does it cite the current literature?

Yes

If applicable, is the statistical analysis and its interpretation appropriate?

Not applicable

Are all the source data underlying the results available to ensure full reproducibility?

Yes

Is the study design appropriate and is the work technically sound?

Yes

Are the conclusions drawn adequately supported by the results?

Yes

Are sufficient details of methods and analysis provided to allow replication by others?

Yes

Reviewer Expertise:

Referee suggested by the NC3Rs for their scientific expertise and experience in assessing 3Rs impact.

I confirm that I have read this submission and believe that I have an appropriate level of expertise to confirm that it is of an acceptable scientific standard.
